# Empagliflozin and Right-Sided Heart Failure: A Comprehensive Review of Emerging Evidence and Clinical Perspectives

**DOI:** 10.7759/cureus.84064

**Published:** 2025-05-13

**Authors:** Raj K Chaudhary, Obaid Ali, Amrendra Kumar, Abilesh Kumar, Syed Mustafizur Rab

**Affiliations:** 1 Department of Medicine, Jawaharlal Nehru Medical College, Bhagalpur, IND; 2 Department of Medicine, Mahatma Gandhi Mission (MGM) Medical College and Hospital, Aurangabad, IND

**Keywords:** cardiometabolic therapy, empagliflozin, hemodynamic modulation, right-sided heart failure, right ventricular mechanics

## Abstract

Right-sided heart failure (RHF) remains a clinically challenging and under-researched condition often managed through extrapolated guidance from left-sided heart failure data. This review explores the therapeutic promise of empagliflozin, a sodium-glucose co-transporter 2 inhibitor, for RHF management. While empagliflozin is currently approved for glycemic control and heart failure with reduced ejection fraction, its potential to improve symptoms, functional capacity, and cardiovascular outcomes in RHF warrants focused investigation. In the absence of RHF-specific randomized controlled trials (RCTs), this review synthesizes insights from preclinical models, subgroup analyses, and surrogate markers drawn from major trials such as EMPEROR-Reduced, EMPEROR-Preserved, and EMPIRE-HF. Empagliflozin’s mechanisms are conceptually grouped as hemodynamic, reducing preload, afterload, and venous congestion, and improving right ventricle-pulmonary artery coupling and metabolic, enhancing myocardial energetics, reducing inflammation and fibrosis, and inhibiting the Na⁺/H⁺ exchanger (NHE1). Although RHF patients were not separately stratified in these trials, indirect benefits observed through TAPSE improvement, renal protection, and congestion relief support further exploration. This review emphasizes the need for RHF-specific RCTs, mechanistic studies, and real-world cohorts to validate and expand empagliflozin’s therapeutic scope. Overall, empagliflozin emerges as a mechanistically sound and clinically promising candidate for transforming the management of RHF by targeting both cardiac and renal dysfunction.

## Introduction and background

Heart failure affects over 64 million individuals globally and remains a formidable clinical challenge with high mortality and escalating healthcare expenditures. Historically, the focus of research and therapeutic development has been on left-sided heart failure (LHF), particularly heart failure with reduced ejection fraction (HFrEF), where pathophysiological understanding has translated into improved outcomes. In contrast, right-sided heart failure (RHF), whether as a primary condition or secondary to pulmonary hypertension or left-sided failure, remains underexplored in both mechanistic and interventional studies. This insufficiency stems from a dual gap, i.e., the absence of large-scale randomized controlled trials (RCTs) and an incomplete understanding of the pathophysiology specific to the right ventricle. RHF, characterized by impaired right ventricular (RV) output leading to systemic venous congestion, hepatic dysfunction, peripheral edema, and ascites, is associated with poor prognosis and lacks therapies targeted specifically at RV dysfunction. Existing treatments are largely extrapolated from LHF trials, despite differing ventricular physiology and response to pharmacologic agents [1,2].

Sodium-glucose co-transporter 2 (SGLT2) inhibitors have emerged as a novel class of drugs in heart failure management, originally developed for glycemic control in type 2 diabetes. Among these, empagliflozin has shown consistent cardioprotective and renoprotective effects across diverse patient populations. While it is premature to definitively label it as a “top agent” for RHF, emerging consensus, based on trial data from EMPA-REG OUTCOME [3], EMPEROR-Reduced [4], and EMPEROR-Preserved [5], indicates a favorable therapeutic profile in heart failure across the ejection fraction spectrum. These effects include reduced cardiovascular mortality and hospitalization, observed in both diabetic and non-diabetic patients.

Importantly, recent data suggest that empagliflozin may benefit the RV structurally and functionally, despite most trials focusing on left ventricular (LV) endpoints [6]. In the EMPIRE-HF trial, empagliflozin was associated with improvements in right ventricular ejection fraction (RVEF) and tricuspid annular plane systolic excursion (TAPSE), both clinically relevant markers of RV systolic function [7]. These benefits can be broadly categorized into three interrelated physiological domains. From a hemodynamic perspective, empagliflozin reduces both preload and afterload, thereby lowering RV wall stress and improving cardiac efficiency. On the metabolic front, the drug enhances myocardial energetics by improving ATP production efficiency, which is particularly beneficial for the energetically compromised RV myocardium. Lastly, at the molecular level, empagliflozin exerts anti-inflammatory effects and reduces interstitial fibrosis, mechanisms thought to be mediated in part through microRNA regulation [8,9].

Although the EMPA-HEART CardioLink-6 trial primarily demonstrated a reduction in LV mass and myocardial fibrosis [10], these findings are indirectly relevant to RV function. Reduced LV filling pressures may attenuate pulmonary vascular resistance, thereby relieving RV afterload. This extrapolation, however, must be acknowledged with caution, given the trial’s LV-centric design. Nonetheless, the rationale for the RV benefit is mechanistically sound and warrants further dedicated investigation.

Furthermore, the EMPERIAL-Reduced and EMPERIAL-Preserved trials highlighted functional improvements in exercise capacity, an outcome highly dependent on RV performance [11]. Empagliflozin’s diuretic-sparing natriuretic effect also supports favorable cardiorenal interactions, crucial in managing volume overload in RHF [12].

**Figure 1:**
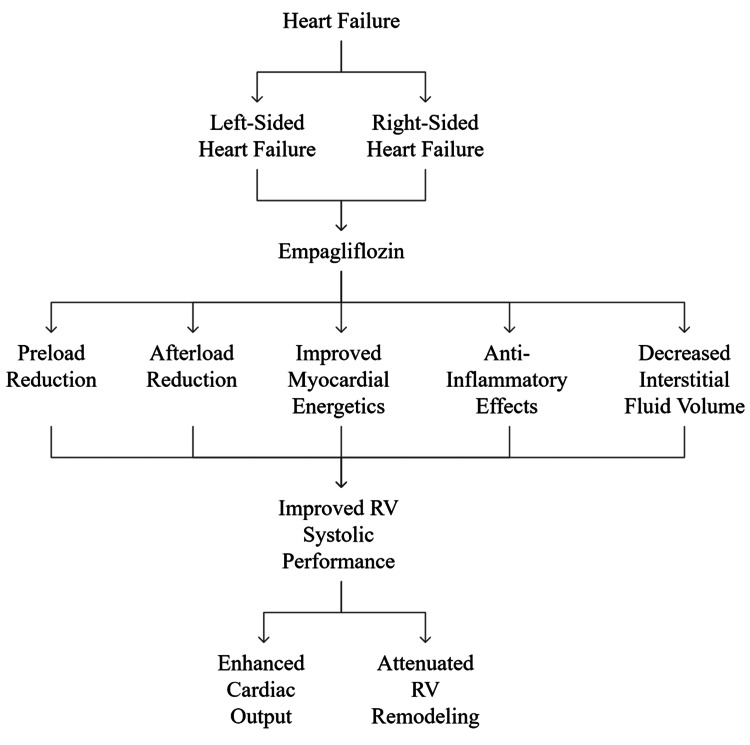
Schematic representation of the multifaceted role of empagliflozin in heart failure. RV: right ventricular Image credit: Raj Kamal Chaudhary

Given this multidimensional evidence, empagliflozin appears to hold potential in altering the course of RHF. This review aims to critically appraise the mechanistic, experimental, and early clinical evidence that supports its expanding therapeutic role (Figure 1).

Objectives of the review

In light of these evolving insights, this review critically examines and synthesizes current research on the therapeutic role of empagliflozin in RHF, an area that has historically received limited direct clinical focus. Beyond summarizing foundational and emerging evidence, the review uniquely aims to address specific clinical questions, such as can empagliflozin modulate RV systolic function independently of its effects on left-sided parameters? What are the drug’s quantifiable effects on RV-specific markers such as TAPSE, RVEF, and systemic venous congestion? Does it influence cardiorenal dynamics differently in RHF compared to LHF? Drawing from mechanistic studies, post hoc analyses of major trials, and preclinical models, this review highlights how empagliflozin may exert therapeutic benefits through hemodynamic unloading, renal optimization, and myocardial remodeling specifically within the right heart context. Hence, it not only evaluates the drug’s translational potential for RHF management but also outlines research gaps, such as the lack of RHF-focused RCTs and RV-specific endpoints, that must be addressed to guide future clinical trials and real-world applications.

## Review

Methodology

A structured search of Web of Science, Scopus, and PubMed databases was conducted to identify relevant literature for this review. Search terms included “empagliflozin” in combination with “right-sided heart failure,” “right ventricular dysfunction,” “SGLT2 inhibitors,” “pulmonary hypertension,” and “cardiorenal syndrome,” covering both clinical and mechanistic domains. The review included only English-language, peer-reviewed publications from the past 10-12 years, which introduces potential limitations due to language restriction and the exclusion of older or non-English studies. Selection criteria prioritized RCTs, high-quality observational studies, and translational research that investigated the pharmacological mechanisms or therapeutic outcomes of empagliflozin in heart failure. Preclinical animal studies were also included when they provided relevant mechanistic insights, though these findings often lack real-world human validation and therefore offer limited generalizability to heterogeneous clinical populations.

Clinical and pathophysiological insights into right-sided heart failure

RHF presents a diagnostic and therapeutic challenge that is often under-recognized in clinical practice. One major reason for missed diagnosis is the overlap of RHF symptoms, such as dyspnea, fatigue, and edema, with those of LHF, making differential diagnosis difficult. Additionally, routine echocardiographic assessments frequently focus on LV function, while RV evaluation is often limited or omitted, contributing to under-detection. RHF may arise from a range of etiologies, broadly categorized as primary (e.g., pulmonary hypertension, congenital heart disease, chronic lung disease), valvular (e.g., tricuspid regurgitation), or secondary to LHF. It disproportionately affects patients with pulmonary hypertension, elderly populations, and individuals with congenital structural anomalies. A key pathophysiological feature in RHF is RV-pulmonary artery (PA) coupling dysfunction, where the failing RV cannot effectively adapt to increased afterload imposed by elevated PA pressures. This mismatch accelerates RV dilation and systolic failure. The disease trajectory typically begins with elevated pulmonary vascular resistance, leading to RV pressure overload, subsequent right atrial and systemic venous congestion, and ultimately impaired forward flow. This cascade results in hepatic congestion, renal dysfunction, and reduced exercise tolerance, driven by neurohormonal activation and impaired organ perfusion. Importantly, the structurally thin-walled RV is more susceptible than the LV to pressure-induced damage, especially in conditions such as pulmonary hypertension and chronic thromboembolic pulmonary disease. Emerging evidence also implicates genetic factors and molecular mechanisms, including dilated cardiomyopathy mutations and microRNA-mediated remodeling, in RV dysfunction and maladaptation [13-17]. Given the mechanistic divergence from LHF, future treatment strategies must extend beyond pharmacologic repurposing and explore targeted molecular therapies and potentially device-based interventions that specifically address RHF pathophysiology.

Diagnostic and prognostic challenges in right ventricular dysfunction

The clinical detection of RHF remains exceptionally challenging due to the absence of standardized diagnostic criteria and the limited reliability of conventional biomarkers and imaging tools in this context. Natriuretic peptides, while commonly used, may be confounded by coexisting hepatic or renal dysfunction, both of which are prevalent in RHF and can distort biomarker accuracy. Similarly, ejection fraction predominantly reflects LV function, offering little insight into RV performance. As a result, RHF diagnosis often requires a multimodal approach that incorporates various echocardiographic parameters. Among these, right ventricular fractional area change (RVFAC) and TAPSE are commonly used. However, RVFAC is highly load-dependent, and TAPSE reflects only longitudinal RV motion, omitting radial and global functional components. Additionally, the crescentic shape and anterior orientation of the RV compromise the geometric assumptions used in standard two-dimensional echocardiographic measurements, making accurate volumetric analysis difficult.

While advanced imaging modalities such as cardiac MRI and RV strain imaging offer more precise assessments, they remain cost-prohibitive and are often unavailable in non-specialist or resource-limited settings, leading to underdiagnosis and misclassification of RHF. Clinical signs such as jugular venous distention, peripheral edema, and ascites further complicate detection due to their symptomatic overlap with hepatic and renal disorders. Importantly, RV dysfunction is associated with increased mortality and hospitalization rates in both heart failure with preserved ejection fraction (HFpEF) and HFrEF populations [13,14,18]. Despite this, major clinical trials have historically underrepresented right heart-specific endpoints, frequently omitting parameters such as TAPSE or RVFAC, and management guidelines continue to focus predominantly on LHF. These diagnostic gaps and prognostic blind spots reinforce the urgent need for RV-focused clinical studies and evidence-based RHF treatment strategies.

Therapeutic gaps in managing right-sided heart failure

The prevailing strategies for RHF management have traditionally focused on symptomatic relief, primarily through diuretics to reduce venous congestion and peripheral edema. However, patients with RHF are particularly vulnerable to volume shifts, and even small changes in preload or afterload can precipitate hemodynamic deterioration, highlighting the precarious balance in managing these patients [19]. Although diuretics remain a mainstay, their long-term efficacy is limited by resistance, electrolyte imbalances, and declining renal function.

The pharmacologic landscape for RHF remains underdeveloped. While guideline-directed therapies such as beta-blockers, mineralocorticoid receptor antagonists, and angiotensin receptor-neprilysin inhibitors have demonstrated efficacy in LHF, their benefit in RHF remains unclear, often not because of proven inefficacy but due to the exclusion or underrepresentation of RHF patients in landmark trials [13,20]. Furthermore, RV-specific endpoints such as TAPSE or RVFAC are seldom assessed, further limiting insights into RV-targeted outcomes. The resulting clinical gap is not due to withheld treatments but to the absence of therapies specifically developed for RHF pathophysiology (Table 1).

**Table 1:** Key pharmacological strategies in right-sided heart failure and their underlying mode of action. RV: right ventricular; LV: left ventricular; RHF: right-sided heart failure; PDE-5: phosphodiesterase-5; SGLT2: sodium-glucose co-transporter 2

Therapy class	Mode of action	Typical clinical focus in RHF	References
Loop diuretics	Interfere with solute reabsorption in the loop of Henle’s thick ascending loop, promoting natriuresis and diuresis	Used to manage volume overload, reduce peripheral edema, and relieve venous congestion	[19]
Beta-blockers	Inhibit beta-adrenergic receptors, lowering heart rate and myocardial oxygen requirement	Applied in selected cases of RV dysfunction, especially with arrhythmias or tachycardia	[13]
Pulmonary vasodilators (e.g., PDE-5 inhibitors)	Increase cyclic GMP in pulmonary vascular smooth muscle, leading to vasodilation	Employed in RHF with associated pulmonary arterial hypertension to reduce RV afterload	[16]
Mineralocorticoid receptor antagonists	Block aldosterone receptors, reduce myocardial fibrosis, and sodium retention	Utilized in RHF with systemic congestion, particularly in patients with coexistent LV dysfunction	[2]
Angiotensin receptor–neprilysin inhibitors	Hinder angiotensin receptors and inhibit neprilysin to enhance natriuretic peptides	Occasionally used in RHF with biventricular failure; extrapolated from LHF data	[20]
SGLT2 inhibitors (e.g., empagliflozin)	Promote glucose and sodium excretion, reduce preload/afterload, improve myocardial energetics, and renal perfusion	Emerging therapy for RHF due to multifactorial effects on volume, neurohormonal activity, and RV-pulmonary coupling	[21]

Moreover, RHF is not a homogeneous condition, and management should be individualized based on the underlying etiology (e.g., pulmonary arterial hypertension, chronic lung disease, secondary to LHF) and hemodynamic profile. Treatment outcomes have often been conflicting, especially with pulmonary vasodilators and endothelin receptor antagonists, which show limited efficacy in non-pulmonary arterial hypertension RHF populations or cause adverse effects due to altered preload sensitivity [16].

Amid this complexity, SGLT2 inhibitors such as empagliflozin have emerged as promising candidates, representing a paradigm shift from purely symptomatic approaches to potentially disease-modifying therapies. Their multifaceted effects, such as hemodynamic optimization, renal protection, and metabolic regulation, make them well-suited for addressing multiple RHF pathophysiological targets [21,22].

SGLT2 inhibitors as cardiovascular therapies in heart failure

Although SGLT2 inhibitors were initially introduced for glycemic control in type 2 diabetes, they have since demonstrated significant cardiovascular and renal benefits. Their effectiveness in heart failure is largely due to glucose-independent mechanisms, which can be categorized into hemodynamic and metabolic domains. Hemodynamically, SGLT2 inhibitors promote osmotic diuresis and natriuresis, leading to reduced preload and afterload without the adverse effects often seen with loop diuretics, such as hypokalemia, neurohormonal activation, or diuretic resistance. This makes them especially attractive in RHF, where patients are particularly sensitive to volume shifts.

Metabolically, the “fuel hypothesis” explains how SGLT2 inhibitors promote a shift in myocardial substrate utilization from fatty acids and glucose to ketone bodies, which are more oxygen-efficient and generate more ATP per unit of oxygen consumed. This is particularly relevant in heart failure, where the myocardium faces an energy deficit. In the EMPEROR-Reduced and DAPA-HF trials, empagliflozin and dapagliflozin significantly reduced heart failure hospitalizations and cardiovascular mortality, regardless of diabetes status, reinforcing their role as disease-modifying agents [21-24].

Empagliflozin exerts its effects through the following three primary mechanisms: (1) preload and afterload modulation via diuretic-like effects and blood pressure reduction; (2) ionic transport regulation, notably through inhibition of the cardiac Na⁺/H⁺ exchanger, which lowers intracellular sodium and calcium levels, improving myocardial relaxation; and (3) cellular signaling modulation, including enhanced mitochondrial efficiency and anti-inflammatory actions. These pathways are particularly important in RV failure, where ATP depletion due to pressure overload and hypoperfusion is prominent.

Additionally, autophagy, the process of cellular debris clearance and energy recycling, plays a critical role in preserving cardiomyocyte health under stress. In RHF, especially under conditions of RV-PA uncoupling, impaired autophagy may exacerbate dysfunction. SGLT2 inhibitors may support autophagic activity, thereby contributing to myocardial resilience in the RV. Taken together, these mechanisms suggest that empagliflozin offers targeted advantages for RHF beyond traditional therapies and warrant further dedicated investigation in RV-specific contexts.

Pharmacological mechanisms of empagliflozin in cardiac function

The primary mechanism of action for empagliflozin as an SGLT2 inhibitor involves decreasing renal glucose reuptake in proximal tubules. The cardiovascular advantages of empagliflozin surpass glycemic control benefits through various mechanisms that benefit both LHF and RHF patients. Empagliflozin demonstrates three key actions on cardiac function through hemodynamic mechanisms and metabolic processes, and cellular effects that work together [23,24]. The primary effect of empagliflozin causes both natriuresis and osmotic diuretics, resulting in moderate plasma volume reduction and subsequent preload and afterload decrease. These alterations help manage volume-overloaded states by reducing stress on both ventricles that results from pressure overload [25]. The Na+/H+ exchanger (NHE1) gets inhibited by empagliflozin at the myocardial level, which results in lower sodium and calcium levels inside cells. The improved calcium management helps the heart relax better while potentially reducing fibrosis development [26].

Empagliflozin elevated expression levels of autophagy-related genes and reduced pro-inflammatory responses in cardiac cells [27]. The molecular adaptations demonstrate specific advantages for RV failure because the pathophysiological elements include maladaptive remodeling, together with energy starvation and inflammation. According to the “fuel hypothesis,” the myocardial shift toward more efficient ketone body utilization improves energy metabolism in both ventricles [20].

**Figure 2:**
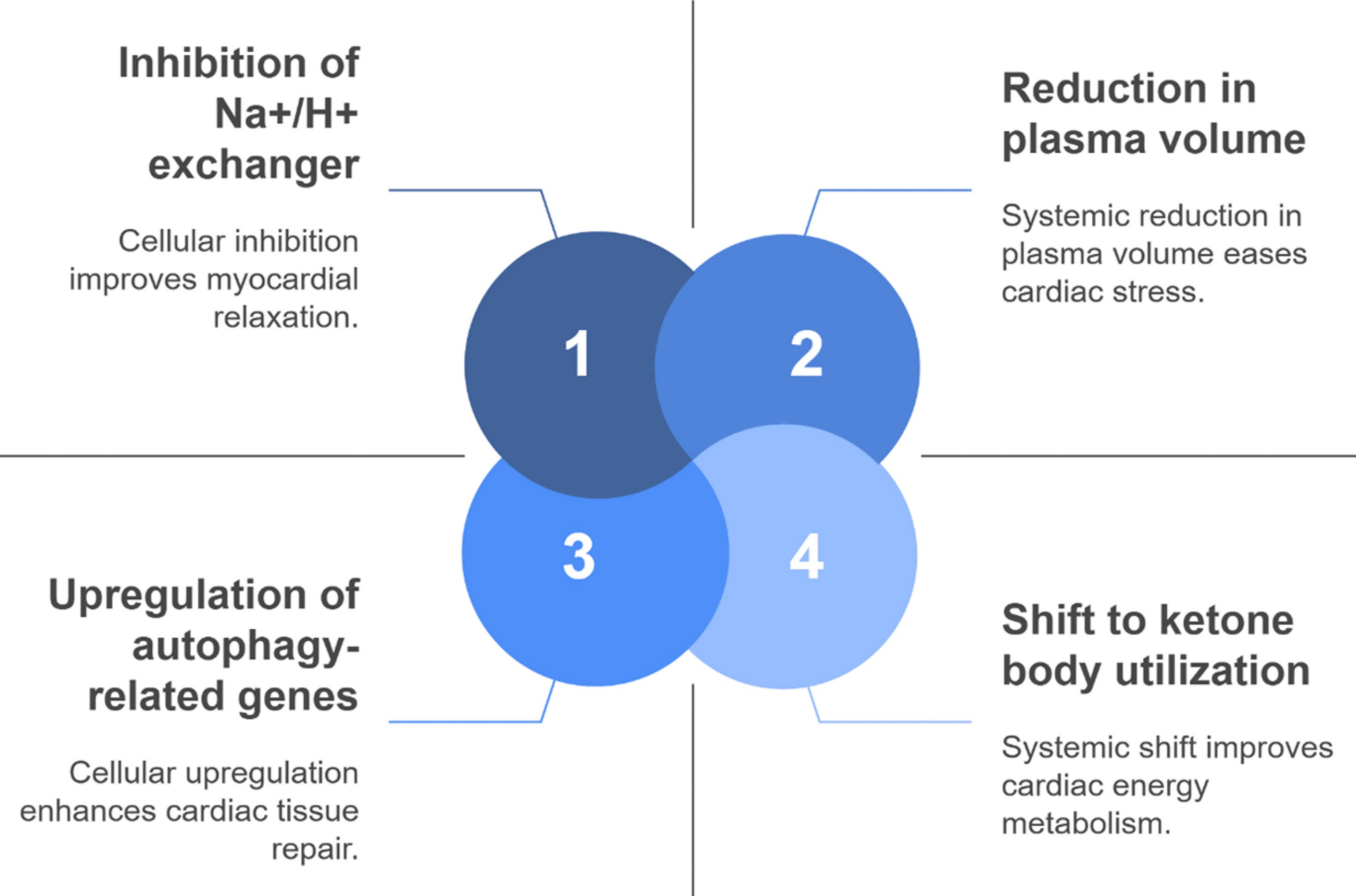
Key mechanistic actions of empagliflozin in heart failure. Image credit: Raj Kamal Chaudhary

Consolidated therapeutic benefits from empagliflozin treatment include a reduction of sympathetic nervous system activation along with a decrease in inflammatory cytokines at the systemic level. The properties of empagliflozin work to decrease interstitial fluid and venous congestion, which helps prevent hepatic and renal impairment commonly observed in RHF [28]. Multiple biological pathways explain how empagliflozin establishes its position as a key medicinal ingredient for the treatment of heart failure. The synergistic mechanisms of empagliflozin support RHF by ionic modulation, enhanced cardiac metabolism, autophagy induction, and plasma volume reduction, as shown in Figure 2.

Hemodynamic effects of empagliflozin on right heart circulation

Proper modulation of hemodynamic conditions is essential in managing RHF, as the RV’s thin-walled structure makes it highly susceptible to afterload changes. Elevated pulmonary vascular resistance often precipitates RV failure, making interventions that reduce pulmonary pressures without compromising systemic perfusion especially valuable. In patients with HFrEF, empagliflozin has been shown to lower pulmonary capillary wedge pressure and increase cardiac output without inducing hypotension, indicating favorable hemodynamic effects [29]. While these findings stem from LHF populations, they provide a mechanistic rationale that may extend to RHF, particularly given the interdependence of ventricular function and the shared hemodynamic burden in biventricular failure. However, direct evidence of these effects in RHF populations remains limited, and further investigation is warranted. These benefits align with RHF management goals, as empagliflozin supports both preload and afterload reduction through a mechanism distinct from traditional diuretics. In the EMPIRE-HF trial, empagliflozin was associated with improved RV performance based on TAPSE and RV longitudinal strain measurements [30]. While these metrics are useful indicators of RV-PA coupling and systolic function, it is important to note that both are load-dependent and may not fully capture global RV function, particularly under fluctuating hemodynamic conditions.

Although some findings suggest that empagliflozin may influence pulmonary vascular tone, this remains a hypothesis under active investigation, with current support stemming from observations of its anti-inflammatory and anti-proliferative effects [31]. These actions may contribute indirectly to afterload reduction, particularly in RHF patients with pulmonary hypertension, but direct mechanistic evidence remains limited. Empagliflozin also facilitates a form of physiological decongestion, characterized by gradual interstitial fluid removal with minimal activation of the renin-angiotensin-aldosterone system (RAAS) and lower risk of intravascular volume depletion. This contrasts with the more abrupt fluid shifts induced by loop diuretics, which, while essential in acute decompensation, can provoke electrolyte imbalances and neurohormonal activation. Furthermore, empagliflozin has been shown to enhance the efficacy of loop diuretics, likely through improved natriuresis and reduced diuretic dose requirements, thereby promoting more stable fluid management. While loop diuretics remain indispensable in acute RHF settings for rapid decongestion, they do not modify the disease course. Empagliflozin, in contrast, offers broader cardiovascular and renal benefits, making it a potential disease-modifying therapy for RHF. Its multifaceted mechanisms, which span hemodynamic modulation and myocardial metabolic support, highlight a shift in RHF management from short-term symptom control toward long-term pathophysiological intervention.

Dual organ benefits of empagliflozin in heart failure: a cardiorenal perspective

The pathophysiology of RHF depends on heart-kidney interactions because venous congestion and reduced renal perfusion produce a continuous cycle that worsens volume overload and renal dysfunction. The intricate nature of this relationship proves challenging to control, especially when standard diuretic treatments lose their effectiveness. Empagliflozin demonstrates a strong potential as a therapeutic choice because it improves cardiac and renal pathway functions [25]. Empagliflozin uses osmotic diuresis and natriuresis to reduce plasma volume, yet avoids RAAS activation to the same degree as loop diuretics do. Empagliflozin produces a physiological decongestion process that prevents both significant fluid volume shifts and electrolyte imbalances, which remain crucial for RHF patients who need precise preload management [23].

Clinical research from the RECEDE-CHF trial showed that empagliflozin enhanced loop diuretic performance while maintaining kidney function when used with loop diuretics [32]. Empagliflozin demonstrated its ability to decrease the required loop diuretic dose while protecting against renal tubular injury [32]. The combination of benefits creates an optimal renal-friendly volume-management approach that is suitable for treating RHF. The DAPA-CKD trial analyzed dapagliflozin while establishing substantial reductions of renal illness in its latter stages of development and cardiovascular death in patients without diabetes [33]. The renal outcomes from EMPEROR-Reduced and EMPEROR-Preserved trials show identical results, which supports the notion that these cardiorenal benefits stem from a class effect [21,34]. Table 2 shows that empagliflozin provides multiple advantages beyond its diuretic function by protecting renal function and improving heart structure and patient well-being, but loop diuretics serve only as decongestive agents without disease-changing capabilities.

**Table 2:** Distinct clinical and physiological features of empagliflozin and loop diuretics in managing right-sided heart failure. The table is based primarily on evidence from HFrEF or general heart failure populations. While extrapolation to RHF is mechanistically plausible, RHF-specific trial data remain limited. SGLT2: sodium-glucose co-transporter 2; RAAS: renin-angiotensin-aldosterone system; MRA: mineralocorticoid receptor antagonist; KCCQ: Kansas City Cardiomyopathy Questionnaire; HF: heart failure; RHF: right-sided heart failure; HFrEF: heart failure with reduced ejection fraction

Parameter	Empagliflozin	Loop diuretics	References
Mechanism of action	Inhibits SGLT2 in proximal renal tubules, boosting glucose excretion (osmotic diuresis and natriuresis)	Inhibits Na⁺/K⁺/2Cl⁻ transporter in the loop of Henle, causing potent diuresis	[22]
Neurohormonal activation	Minimal activation of the RAAS or the sympathetic system	Activates RAAS and the sympathetic nervous system, especially at high doses	[19]
Hemodynamic profile	Reduces preload and afterload gently; improves right ventricle-pulmonary artery coupling	Primarily reduces preload through aggressive volume depletion	[12]
Effect on renal function	Preserves renal perfusion and function even during decongestion	Risk of renal impairment due to intravascular volume depletion	[27]
Electrolyte stability	Mild risk of hypovolemia; preserves serum potassium	High risk of electrolyte disturbances (hypokalemia, hypomagnesemia)	[11]
Diuretic synergy	Enhances the effectiveness of loop diuretics when used concurrently	Often requires a combination with thiazides or MRAs to overcome resistance	[32]
Impact on inflammation and fibrosis	Reduces pro-inflammatory cytokines; inhibits myocardial fibrosis pathways	No known anti-inflammatory or anti-fibrotic effects	[30]
Effect on functional capacity	Improves exercise tolerance and quality of life (KCCQ score improvements)	Reduces congestion but has no proven prolonged impact on functional capacity	[18]
Mortality benefit	Associated with a diminution in hospitalizations for HF and cardiovascular death in trials	No independent mortality benefit established	[4]

Mechanistic and clinical insights on the cardiovascular benefits of empagliflozin in the right-sided heart failure context

Preclinical studies have provided foundational insights into the cardiovascular mechanisms of empagliflozin. In a non-diabetic rat model of myocardial infarction with LV dysfunction, empagliflozin treatment was associated with improved biventricular function, including RV performance [8]. However, it is important to clarify that the RV benefits may have resulted secondarily from LV unloading, and the independent effect on RV function has not been isolated in RV-specific failure models. At the molecular level, empagliflozin modulates gene expression related to mitochondrial energy metabolism and oxidative phosphorylation, potentially enhancing ATP production and myocardial efficiency, which is particularly relevant under RV pressure overload conditions [16]. Though promising, specific RV-targeted mitochondrial biomarkers or ATP yield measurements remain sparse. Inhibitory action on the cardiac Na⁺/H⁺ exchanger (NHE1) further contributes to intracellular sodium and calcium balance, promoting improved diastolic relaxation. However, the proposed anti-arrhythmogenic effect from this mechanism is inferred and not yet validated through RV-specific electrophysiological models.

Empagliflozin has also demonstrated anti-fibrotic and endothelial-stabilizing properties in LV myocardial tissue and systemic vasculature, with some evidence in pulmonary vascular beds, which may support improved pulmonary hemodynamics in RHF contexts. Yet, direct histological confirmation in RV myocardium is currently lacking [35]. Clinical trial data further underscore the broad cardiovascular potential of empagliflozin. The EMPEROR-Reduced and EMPEROR-Preserved trials demonstrated significant reductions in cardiovascular mortality and heart failure hospitalizations across a spectrum of ejection fractions, irrespective of diabetic status [4,34]. While these trials focused primarily on LHF, their findings are clinically relevant to RHF due to overlapping phenotypes such as pulmonary hypertension and diastolic dysfunction. A post hoc sub-analysis of the EMPIRE-HF trial revealed improvements in RV systolic function, evidenced by increased TAPSE and reduced right ventricular systolic pressure (RVSP) [30]. However, these parameters are load-dependent and do not fully capture intrinsic RV contractility, limiting the interpretability of these findings. Moreover, the observed improvements were hypothesis-generating rather than powered for statistical significance. The EMPULSE trial, which investigated early empagliflozin administration in patients hospitalized for acute heart failure, included individuals with suspected RHF. However, RHF-specific stratification was not performed, and conclusions about RHF must be interpreted with caution [36].

Importantly, across trials, empagliflozin has shown a favorable safety profile, including no significant impact on blood pressure in patients with RV dysfunction. Its renal protective effects, along with volume regulation via gradual decongestion and minimal RAAS activation, suggest a potential advantage over traditional loop diuretics for RHF management. These findings support further investigation into empagliflozin as a disease-modifying treatment in RHF. However, dedicated RHF-specific RCTs remain essential to validate these promising observations.

Post hoc and subgroup analyses: emerging right ventricular insights

The RV effects of empagliflozin have primarily been observed in post hoc analyses and subgroup evaluations of larger trials that originally focused on patients with LHF. These exploratory findings highlight the urgent need for studies that incorporate RHF-specific endpoints, which have historically been underrepresented in major cardiovascular trials. In a sub-analysis of the EMPIRE-HF trial, empagliflozin treatment was associated with improvements in TAPSE and RVSP parameters that reflect RV systolic performance and pulmonary hemodynamics [10]. However, both measures are load-dependent, and their ability to assess intrinsic RV contractility remains limited. Moreover, the findings were hypothesis-generating, and the sample size was not powered for RHF-specific outcomes. Subgroup analyses from the EMPEROR-Reduced and EMPEROR-Preserved trials suggested potential benefits in patients with elevated pulmonary pressures, possibly reflecting improved right heart remodeling and symptom relief [34]. Still, these effects were not primary endpoints, and RHF was not independently stratified, limiting the certainty of their applicability to RHF. Similarly, empagliflozin was found to reduce right heart strain in patients with type 2 diabetes in studies not involving overt heart failure [37], indicating a potential preventive cardioprotective effect, although its relevance to established RHF remains speculative.

**Figure 3:**
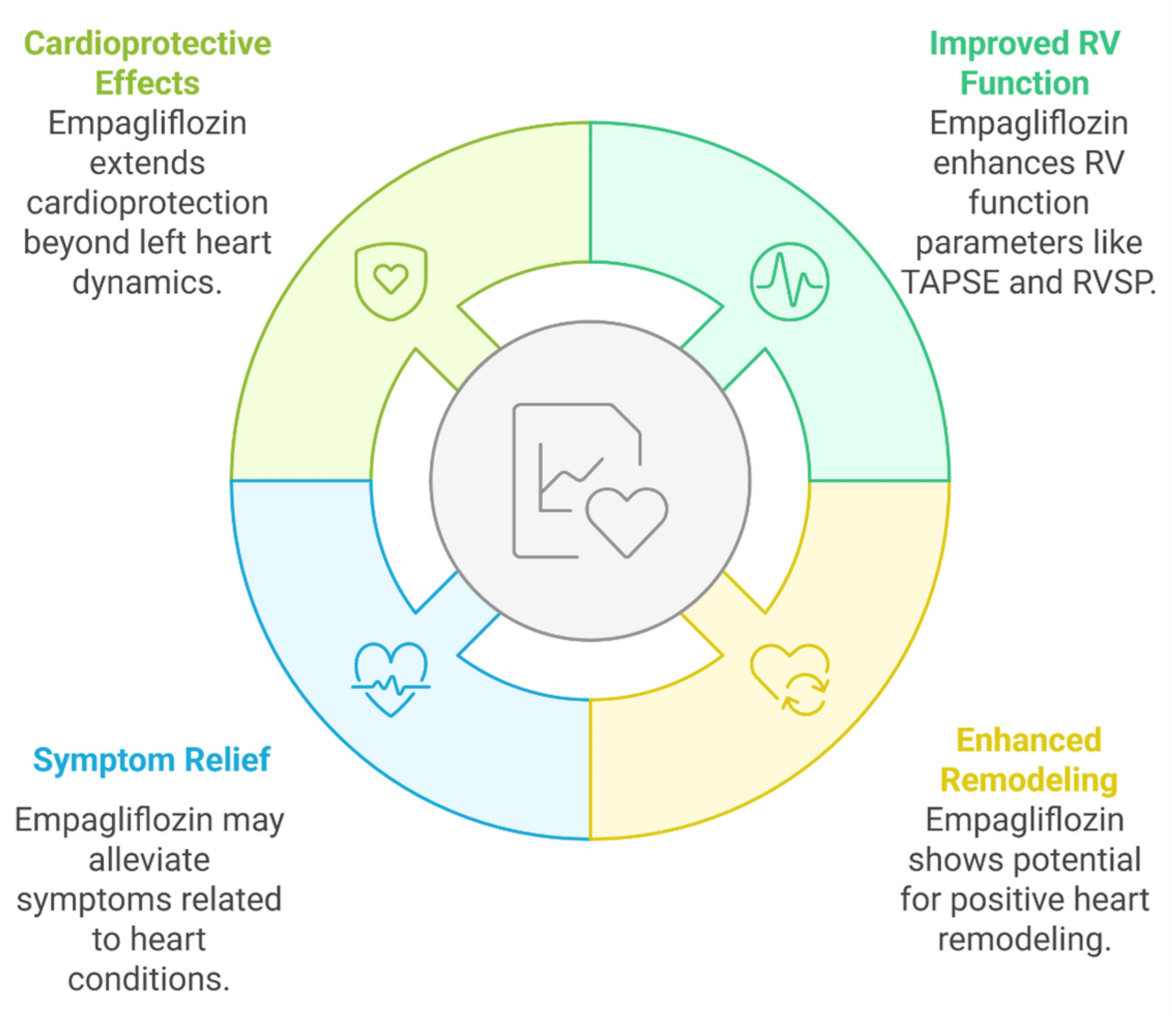
Multidimensional effects of empagliflozin on right ventricular function. RV: right ventricular; RVSP: right ventricular systolic pressure; TAPSE: tricuspid annular plane systolic excursion Image credit: Raj Kamal Chaudhary

The RV recovery effects of empagliflozin are believed to occur through interconnected mechanisms such as anti-inflammatory signaling, inhibition of the cardiac Na⁺/H⁺ exchanger (NHE1), attenuation of fibrosis, and possibly modulation of pulmonary vascular resistance. However, the latter is primarily supported by preclinical and mechanistic models, with limited direct evidence from human studies. These pathways may enhance myocardial relaxation, reduce wall stress, and improve RV-PA coupling, but further investigation is warranted. While SGLT2 inhibitors as a class confer benefits such as natriuresis, improved renal function, and reduced ventricular preload, potential agent-specific differences, including the impact of empagliflozin on mitochondrial energetics and RV pressure dynamics, should be considered in future studies. It is, therefore, premature to declare empagliflozin as a foundational therapy for RHF. Instead, it should be viewed as a promising adjunctive therapy, with further validation required through prospective, RHF-specific RCTs. The right heart recovery benefits of empagliflozin appear through multiple interconnected pathways that support myocardial mechanics while providing structural support and patient-centered clinical outcomes, as illustrated in Figure 3.

Empagliflozin in the spectrum of SGLT2 inhibitors for right ventricular dysfunction

The SGLT2 inhibitor class of medications, including empagliflozin, dapagliflozin, and canagliflozin, has substantially advanced heart failure therapy. The pharmacologic differences between agents remain minor, yet research shows that the entire class produces similar cardiorenal effects. The evaluation of RHF applications requires a separate assessment of observations at the class level from those specific to individual drugs.

The major cardiac and renal outcomes in the DAPA-HF trial using dapagliflozin matched the results of the EMPEROR trials, which used empagliflozin. Post-hoc data from these studies showed that right heart pressure elevation and venous congestion signs benefited from SGLT2 inhibitor treatment, despite RHF endpoints not being the main study focus. These findings suggest potential right heart benefits from SGLT2 inhibitors.

Evidence from a meta-analysis supports the role of SGLT2 inhibitors in lowering pulmonary vascular resistance and cardiovascular events, potentially benefiting RV function [38]. Additionally, SGLT2 inhibitors may attenuate pulmonary vascular remodeling and thereby reduce RV afterload, a key factor in RHF pathophysiology [31]. The secondary effects of SGLT2 inhibitors differ between each medication. Empagliflozin offers unique myocardial benefits through its inhibition of cardiac sodium-hydrogen exchange that improves intracellular calcium regulation and myocardial relaxation [26]. These properties would specifically benefit drug treatment in conditions that cause right-sided pressure overload. RV failure therapy must rely on SGLT2 inhibitors as a base while keeping in mind the individual cardiometabolic properties of empagliflozin because these details are vital for treatment planning. The unique qualities of empagliflozin distinguish it from other SGLT2 inhibitors because it demonstrates direct evidence of RV function and myocardial sodium handling, and pulmonary vascular modulation, which makes it an especially promising treatment for RHF management (Table 3).

**Table 3:** Comparative evaluation of empagliflozin and other SGLT2 inhibitors in heart failure with emphasis on right-sided involvement. EF: ejection fraction; HFpEF: heart failure with preserved ejection fraction; RV: right ventricular; TAPSE: tricuspid annular plane systolic excursion; KCCQ: Kansas City Cardiomyopathy Questionnaire

Feature	Empagliflozin	Dapagliflozin	Canagliflozin	References
Primary trials in heart failure	EMPEROR-Reduced, EMPEROR-Preserved, EMPULSE	DAPA-HF, DAPA-CKD	CANVAS Program	[21,34]
Efficacy in preserved EF	Proven efficacy in HFpEF with RV overlap	Moderate efficacy; fewer HFpEF-specific insights	Limited data in preserved EF populations	[8]
Direct evidence of RV function	Sub-study data show improvements in TAPSE, RV strain, and RV systolic pressure	No published RV-specific trials	No RV-specific clinical evidence	[29]
Effect on pulmonary vascular remodeling	Proposed anti-remodeling effects through inflammation and fibrosis modulation	Lacks direct data	Not studied	[13]
Na⁺/H⁺ exchanger (NHE1) inhibition	Demonstrated inhibition, contributing to reduced intracellular Na⁺ and Ca²⁺ and improved RV relaxation	Not confirmed	Unclear	[29,32]
Renal-protective effects	Strong and consistent across trials	Strong across trials	Moderate; concerns over amputation and fracture risk	[15]
Effect on functional capacity	Improvements in KCCQ scores and the six-minute walk distance (EMPERIAL trials)	Functional gains in HFrEF, less studied in preserved EF	No reported impact on functional capacity	[3]
Hospitalization and mortality benefit	Significant reductions in mitigating HF hospitalization and cardiovascular death	Comparable benefit in HFrEF	Shown in diabetics, the cardiovascular benefit in HF is less robust	[17]

Clinical implications of empagliflozin for symptom relief and functional improvement in right-sided heart failure

Management of RHF primarily targets symptom control and functional improvement, as RV dysfunction contributes significantly to exertional intolerance and diminished quality of life. RV failure leads to impaired pulmonary perfusion and systemic venous congestion, which together result in inadequate oxygen delivery, dyspnea, fatigue, and reduced exercise capacity [4]. Empagliflozin has shown promise in this setting, particularly through studies assessing functional performance and patient-reported outcomes. In the EMPERIAL-Reduced and EMPERIAL-Preserved trials, empagliflozin improved the six-minute walk distance and Kansas City Cardiomyopathy Questionnaire (KCCQ) scores, suggesting enhancement in daily activity and quality of life [11]. However, these trials primarily included patients with HFrEF and HFpEF, and did not stratify or assess RV dysfunction as a predefined endpoint, limiting their direct applicability to RHF. Similarly, while the EMPEROR-Preserved trial reported quality of life improvements and reduced hospitalizations in HFpEF patients [30], the relevance to RHF is extrapolated, particularly as RHF is common in HFpEF populations with pulmonary hypertension or RV-PA uncoupling.

The EMPULSE trial, which evaluated empagliflozin in acutely decompensated heart failure, included patients likely to have RHF, but did not specifically assess RHF subgroups or RV endpoints [36]. Thus, current evidence supporting empagliflozin in RHF remains indirect and hypothesis-generating, despite strong mechanistic plausibility and overlapping clinical profiles. Empagliflozin may offer complementary benefits in RHF, particularly in patients with volume overload, venous congestion, and cardiorenal involvement, by reducing pulmonary pressures and improving systemic congestion. However, no head-to-head comparisons exist with loop diuretics, and its value should be interpreted as adjunctive, not superior. Statements suggesting empagliflozin reduces the need to increase diuretic doses are currently based on observational insights and mechanistic reasoning, not direct trial data.

Safe implementation requires careful clinical monitoring, especially in patients with systolic blood pressure <90 mmHg or rapid declines in glomerular filtration rate. A multidisciplinary approach is essential: heart failure specialists for pharmacotherapy optimization, nephrologists for volume and renal monitoring, and primary care or rehabilitation teams for functional assessment and lifestyle guidance. While current findings are promising, dedicated RHF-specific RCTs are urgently needed to validate empagliflozin’s effectiveness and safety in this unique population before its routine use in RHF can be recommended.

Bridging evidence and practice: clinical use of empagliflozin in right-sided heart failure

A complex method must be used to turn increasing evidence about RHF management into practical applications. Empagliflozin serves as standard treatment in HFrEF according to guidelines, yet RHF lacks specific guidelines for therapy; hence, healthcare providers must base their decisions on broader clinical findings. Empagliflozin shows particular value for RHF patients who experience fluid retention or show diuretic resistance or have renal impairment because conventional treatments typically fail in these cases. Empagliflozin creates a chance to manage multiple pathophysiological conditions at once by improving sodium excretion and venous decongestion while preserving kidney function [39].

The implementation of empagliflozin in a clinical setting requires a thorough evaluation of individual patient characteristics. The management of patients with substantial fluid loss or borderline blood pressure requires continuous observation to prevent dangerous blood pressure drops or over-diuresis because heart failure patients demonstrate sensitivity to changes in preload [40]. The strategic implementation of empagliflozin reduces the necessity to increase diuretic doses and helps maintain renal function and better regulate hemodynamics [41].

The best results require care teams to work together as a multidisciplinary team. Heart failure specialists and nephrologists, together with cardiologists, need to work jointly in developing comprehensive care plans that incorporate empagliflozin specifically for patients with complex RHF conditions affecting systemic congestion and pulmonary hypertension, and kidney function. The current evidence supports the evidence-based implementation of RHF-specific trials, although randomized trials for this patient population remain necessary for the future.

Clinical guidelines and the future role of empagliflozin in right-sided heart failure management

Empagliflozin attained a central role in cardiac treatment strategies upon its inclusion into current heart failure practice guidelines. It was formally endorsed in the 2021 European Society of Cardiology guidelines and the 2022 American Heart Association/American College of Cardiology/Heart Failure Society of America guidelines as a foundational treatment for patients with HFrEF, irrespective of glycemic status [33,39]. These inclusions followed strong outcome data from large-scale trials and growing recognition of the drug’s broad cardiovascular and renal benefits. These guidelines, however, currently lack specific recommendations for RHF, partly due to persistent diagnostic challenges and under-recognition of the condition. Clinicians often overlook RHF because its clinical features, such as edema, fatigue, and dyspnea, overlap with LHF and non-cardiac conditions, and routine assessment of RV function (e.g., TAPSE, RV strain) is inconsistently performed [17]. RHF is commonly viewed as a secondary manifestation of LHF or pulmonary hypertension, yet it also occurs as an independent clinical syndrome in settings such as isolated RV infarction, primary pulmonary arterial hypertension, and congenital heart disease.

To improve RHF management, forthcoming guidelines should incorporate RHF-specific markers such as TAPSE, RV strain, and systemic venous congestion indicators. These would provide a structured framework for initiating treatments such as empagliflozin in appropriate patients and would help bridge the current gap between RHF pathophysiology and evidence-based care models. While observational studies and expert consensus statements support the use of empagliflozin in RHF, especially in patients with pulmonary hypertension, fluid retention, or cardiorenal syndrome, this use remains off-label and based on indirect evidence extrapolated from LHF trials [42]. Therefore, RHF-specific RCTs are necessary to confirm efficacy, establish safety, and expand the drug’s therapeutic scope more definitively. These studies will also help guide phenotype-based approaches to RHF, in which therapies are tailored to individual profiles such as RV strain patterns, pulmonary pressure gradients, or comorbidities such as renal dysfunction and chronic lung disease. While empagliflozin’s potential in RHF is promising, its role must be supported by direct evidence before becoming part of routine RHF management.

Limitations

Despite a rigorous search strategy, several key limitations within the current evidence base were identified. The majority of empagliflozin-related trials, including EMPEROR-Reduced, EMPEROR-Preserved, and EMPIRE-HF, were designed primarily for LHF populations or mixed heart failure conditions, with most excluding patients with isolated RHF. RV endpoints, when reported, were typically secondary or exploratory and varied significantly across studies, with metrics such as TAPSE, RVSP, and RV strain assessed inconsistently. This lack of standardization in RV functional assessment complicates interstudy comparisons and undermines efforts to perform pooled analyses or meta-analyses. Additionally, patient demographics in existing studies were often limited, with underrepresentation of older adults, women, and non-Western populations, further restricting the generalizability of findings to diverse RHF cohorts.

The phrase “preclinical and translational research on different human populations,” as used in previous iterations of this review, refers to the disconnect between promising data from animal models and the current lack of validation in real-world human subjects. To advance the therapeutic role of empagliflozin in RHF, future clinical trials should address these gaps by enrolling patients with isolated RHF, incorporating predefined right heart endpoints such as RV strain and RVFAC, and employing standardized imaging modalities such as echocardiographic strain analysis or cardiac MRI. These methodological improvements will be critical for establishing reproducible, clinically relevant evidence to inform RHF-specific treatment strategies.

Future directions

Future research should employ standardized assessments of RV function, such as strain imaging and right heart catheterization, to enhance diagnostic accuracy and outcome evaluation. However, these tools have practical limitations: strain imaging depends on equipment compatibility and operator expertise, while right heart catheterization is invasive and resource-intensive, limiting its routine use. Investigating how empagliflozin influences pulmonary vascular remodeling and RV-PA coupling is particularly important, as both processes contribute to increased RV afterload and are closely linked to disease progression and poor outcomes in RHF. Clarifying these mechanisms could help establish empagliflozin’s potential as a disease-modifying agent in RHF. In addition, future trials should evaluate drug-drug interactions, safety profiles, and dose adjustments when empagliflozin is used alongside other RHF treatments such as diuretics, vasodilators, or RAAS inhibitors. Outcome measures should include improvements in exercise capacity (e.g., six-minute walk test), functional status (e.g., KCCQ scores), and hemodynamic parameters. Incorporating biomarkers such as N-terminal pro B-type natriuretic peptide and troponins may aid in risk stratification and personalized therapy. Furthermore, leveraging real-world data can complement clinical trials by capturing diverse patient populations and long-term safety outcomes, ultimately refining empagliflozin’s role in RHF management.

## Conclusions

RHF remains underrecognized and inadequately managed, largely due to diagnostic challenges, limited treatment options, and overlap with LHF and pulmonary diseases. Symptoms such as fatigue, peripheral edema, and dyspnea are nonspecific and often misattributed, delaying accurate diagnosis. Additionally, reliance on tools such as strain imaging and right heart catheterization, which are either technically demanding or invasive, limits routine RV assessment. Current therapies, including diuretics and pulmonary vasodilators, provide symptomatic relief but fail to target key mechanisms such as RV remodeling, myocardial fibrosis, and elevated pulmonary vascular resistance. These limitations underscore the need for therapies that directly address RHF pathophysiology. Empagliflozin, an SGLT2 inhibitor initially approved for glycemic control, has emerged as a promising agent with cardioprotective effects that extend beyond glucose regulation. It improves myocardial energy efficiency by enhancing ketone utilization, reduces inflammation and fibrosis, and inhibits the cardiac Na⁺/H⁺ exchanger (NHE1), promoting better intracellular ion homeostasis and diastolic relaxation. Furthermore, empagliflozin supports renal perfusion and blunts maladaptive neurohormonal activation, critical in RHF progression. Although most clinical trials focus on LHF or mixed populations, subgroup and mechanistic analyses suggest that RHF may similarly benefit. This review highlights these multidimensional effects and emphasizes the urgency of conducting RHF-specific RCTs with standardized RV endpoints. Such efforts are essential to validate empagliflozin’s role as a disease-modifying therapy in RHF and to evolve current treatment strategies from symptom control to pathophysiological intervention.
